# Toward personalized interventions for preventing depression in primary care: Qualitative and quantitative findings from the e-predictD pilot study

**DOI:** 10.1371/journal.pone.0355675

**Published:** 2026-09-11

**Authors:** Patricia Moreno-Peral, Henar Campos-Paíno, Alberto Rodríguez-Morejón, Sonia Conejo-Cerón, Antonina Rodríguez-Bayón, María Isabel Ballesta-Rodríguez, Emiliano Rodríguez-Sánchez, Juan Manuel Mendive, Yolanda López del Hoyo, Juan de Dios Luna, Olaya Tamayo, Juan Ángel Bellón

**Affiliations:** 1 Biomedical Research Institute of Málaga (IBIMA Platform Bionand), Málaga, Spain; 2 Chronicity, Primary Care and Health Promotion Research Network (RICAPPS), ISCIII, Madrid, Spain; 3 Department of Personality, Evaluation and Psychological Treatment, University of Malaga (UMA), Malaga, Spain; 4 Centro de Salud San José, Distrito Sanitario Jaén Norte, Servicio Andaluz de Salud (SAS), Linares, Jaén, Spain; 5 Centro de Salud Federico del Castillo, Distrito Sanitario Jaén, Servicio Andaluz de Salud, (SAS) Jaén, Spain; 6 ‘Miguel Armijo’ Health Centre, Castilla and León Health Service (SACyL), Salamanca, Spain; 7 Department of Medicine, University of Salamanca (USAL), Salamanca, Spain; 8 ‘La Mina’ Health Centre, Institut Català de la Salut (ICS), Barcelona, Spain; 9 Instituto de Investigación Sanitaria de Aragón (IISA). Universidad de Zaragoza (UNIZAR), Zaragoza, Spain; 10 Department of Statistics and Operational Research, University of Granada (UGR), Granada, Spain; 11 El Palo Health Centre, Andalusian Health Service (SAS), Málaga, Spain; 12 Department of Public Health and Psychiatry, University of Málaga (UMA), Málaga, Spain; University of Udine: Universita degli Studi di Udine, ITALY

## Abstract

**Background:**

The predictD intervention, delivered by family physicians (FPs), has demonstrated effectiveness and cost-efficiency in preventing depression and anxiety. The e-predictD study aims to design, develop, and evaluate a novel personalized intervention for depression prevention by integrating information and communication technologies (ICTs), risk prediction algorithms, and decision support systems (DSS) for both patients and FPs.

**Objective:**

To evaluate the satisfaction, usability, and acceptability, of a beta version of the e-predictD intervention in primary care settings.

**Methods:**

The e-predictD intervention follows a biopsychosocial approach, including an initial patient-FP interview, specific FP training, and an app. A β-version was tested in a pilot study without a control group over three months. The app integrates a validated depression risk prediction algorithm, decision algorithms, and a monitoring system supporting the DSS. The DSS generates a personalized prevention plan (PPP) from eight intervention modules: physical exercise, social relationships, problem-solving, communication skills, decision-making, assertiveness, sleep improvement, and cognitive restructuring. Patients and FPs discussed the PPP in a 15-minute baseline interview, selecting modules for implementation over three months. Semi-structured interviews gathered feedback. Assessments included depression (PHQ-9), anxiety (GAD-7), quality of life (SF-12), and major depression risk (predictD algorithm).

**Results:**

Six FPs from six Spanish cities enrolled 56 non-depressed patients at moderate-to-high risk of depression; 47 (84%) completed follow-up. The app was used for a median of six days (interquartile range: 1–30). Both FPs and patients expressed satisfaction, leading to incorporated improvements. After three months, significant reductions in major depression risk and anxiety symptoms were observed, alongside improved mental quality of life. However, no significant changes were found in depressive symptoms or physical quality of life.

**Conclusion:**

This pilot study supports the feasibility and acceptability of the e-predictD β-version, despite lower-than-expected app usability. Health improvements were observed, warranting confirmation in a randomized controlled trial.

**Trial registration:**

ClinicalTrials.gov NCT03990792

## Introduction

Over 300 million people in the world suffer depression [1]1. During the COVID-19 pandemic, the prevalence of depressive disorders increased by 27.6% [2]. Depression has been associated with substantial economic costs, not only because of the medical costs that it generates but also because of the loss of productivity associated with this disease [3]. Most suicides occur among people with depression [4]. In terms of quality of life, the burden of depression, measured as years lived with disability (YLD), ranked second in 2019 [5]. Depression is expected to be the leading cause of disease burden in high-income countries by 2030 [6]^.^

Despite effective treatments for depression [7–9,], the depression prevalence has not decreased [10]. Psychological and pharmacological treatments have a limited efficacy [11] and present problems such as low adherence and reduced acceptability [12]. Moreover, depression is a heterogeneous condition, existing treatments fall short, and many people affected do not have access to adequate care [13,14]. In addition to treatments, prevention is a promising approach to reducing the global disease burden of depression [15,16].

Psychological [15], exercise-based [17] and social support-based [18] interventions for the prevention of depression are effective, although their effect sizes were small. However, even though such effectiveness was small, the generalization of preventive interventions to large populations could have a relevant impact. This scalability would be feasible through information and communication technologies (ICTs).

The internet offers easy and inexpensive access to programs that treat or prevent mental disorders. They are anonymous and thus avoid stigmatization and improving accessibility [19]. Furthermore, it enables scaling to large populations.

m-Health has been defined as health practices implemented in mobile devices [20]. Smartphones offer integration into daily life and the ability to provide timely, personalized interventions. Therefore, health interventions carried out on smartphones have great potential for development, implementation and scalability.

Internet-based interventions have a small effect in the prevention of depression [21–24]. However, the effectiveness of this type of intervention is limited and ensuring user adherence is critical [24,25].

Primary health care is an ideal setting for prevention, offering holistic and accessible care [26–29], and family physicians (FPs) are commonly the first point of contact [23]. Despite this, few randomized controlled trials on the prevention of depression have been conducted in primary care and most of the interventions have been implemented by mental health specialists [30].

Our research group developed and validated the predictD algorithm for predicting the onset of major depression at 12 months in primary care attendees, in Europe [31] and Spain [32]. The Spanish algorithm obtained good calibration and discriminative validation [C-index = 0.82 (0.79–0.84)]. From 39 known risk factors for depression, 12 were included in the Spanish prediction model: sex, age, sex*age interaction, education, childhood physical abuse, and lifetime depression, SF-12 physical score, SF-12 mental score, dissatisfaction with unpaid work, number of serious problems in very close persons, dissatisfaction with living together at home, and taking medication for stress, anxiety or depression.

The Spanish predictD algorithm allowed us to develop a tailored intervention based on both intensity (level) and specific (profile) risk of depression for each patient [33]. This intervention, performed by FPs, reduced the incidence of major depression and anxiety disorders by 23% at the 18-month follow-up compared to the usual care [33,34], and it was also cost-effective [35]. However, some barriers were identified by the FPs such as lack of time, workload and training constraints [36].

The integration of internet interventions, and specifically mobile phone interventions, could be a solution to overcoming these barriers in primary care depression prevention.

Hence, we designed the e-predict study [37], the aim of which was to design, develop and evaluate a personalized intervention to prevent the onset of depression based on ICTs, risk prediction algorithms and decision support systems (DSS) for patients and FPs [37]. This article presents the results of the pilot study assessing satisfaction, usability, and acceptability of a β-version of the e-predictD intervention in primary care.

## Methods

### Study design, setting and recruitment

We conducted a pilot study for testing a β-version of the e-predictD-intervention. This pilot study was included in the e-predictD study whose protocol has been published elsewhere [37]. The e-predictD study is a randomized controlled trial which aims to design, develop and evaluate an intervention to prevent the onset of major depression in primary care [37].

The design was a quasi-experimental pilot study without a control group, with 3 months of follow-up, a quantitative before-after assessment, a qualitative evaluation at 3 months using individual face-to-face semi-structured interviews to know the opinion of patients and FPs involved, and a phenomenology approach. We confirm that all ongoing and related trials for this intervention are registered under the registration number NCT03990792.

As this study was designed as a pilot quasi-experimental clinical trial, the sample size was determined according to feasibility and exploratory objectives, including the assessment of potential contextual differences across provinces. Accordingly, the pilot study included one primary care physician from each of the six participating Spanish provinces, with approximately 10 patients per physician, based on availability and accessibility criteria. This design allowed the evaluation of recruitment procedures, intervention implementation, and data collection under conditions comparable to those planned for the definitive trial, as well as the exploration of participants’ experiences with the intervention. This approach is consistent with current methodological recommendations for pilot and feasibility studies, which state that sample size should be justified according to feasibility and estimation objectives rather than formal hypothesis testing (Kunselman, 2024) [38].

Ethics approval for this study was granted by the following ethics committees in each participating Spanish city: Ethics and Research Regional Committee of Malaga, Ethics Committee for Clinical Research of IDIAP Jordi Gol (Barcelona), Ethics Committee for Clinical Research of Aragon (CEICA), Ethics Committee for Clinical Research of Health Area of Salamanca.

FPs were invited to participate in this study if they were not planning to change their place of work in the next 3 months and belonged to primary care centers (PCCs) of six Spanish cities: Malaga, Jaen and Linares in the south, Salamanca in the center-west, Zaragoza in the north and Barcelona in the north-east of Spain.

On each day of patient recruitment by the FPs, they checked all the patients on their appointment lists to see if they met any of the following exclusion criteria, based on the data contained in their primary care electronic health records: age under 18 or over 55 years; inability to understand or speak Spanish; severe mental disorder (psychosis, bipolar, personality disorder or addiction disorder); cognitive impairment; terminal illness; and/or sensory disability (blindness or deafness). On each day of recruitment, the FPs briefly presented the e-predictD study to all those patients who met none of the exclusion criteria. If patients were interested in the study, the FPs referred them to contact a research assistant placed in an adjoining room. Patients who contacted the research assistants received a more extensive explanation of the e-predictD pilot study and had the opportunity to discuss and ask questions about it. The research assistants gave them the consent form to be signed if they wanted to participate in the screening. The research assistants verified whether the participants who signed the informed consent form met the following inclusion criteria: (1) have a smartphone, tablet or computer and will have an internet connection for the next 12 months; (2) will remain in their place of residence in their respective cities for at least the next 9 months; (3) did not have current clinical depression according to the PHQ-9 decision algorithm [39,40]; and (4) had a probability of depression in the next year ≥15% (predictD risk algorithm) [32]. The research assistants used a web application to record the participants’ responses, and these were automatically incorporated into the database along with the rest of the self-completed measures at baseline.

Patients who had no exclusion criteria and met all inclusion criteria were informed in writing by the research assistants about the e-predictD intervention and that it would be tested to prevent depression and implemented online, through their smartphones and/or tablet-computers. Once the patient information and selection process was complete, the research assistants gave the patients the second consent form to be signed if they wanted to participate in the e-predictD pilot study. If this last informed consent was signed, then the research assistants provided the participants with the web address of the e-predictD app and an access code to download the app. In addition, the research assistants were available to help patients install the app on their smartphones if necessary. Participant recruitment for the e-predictD pilot study was conducted from June 4 to October 26, 2018.

### e-predictD Intervention

The e-predictD intervention has been described elsewhere [37]. In brief, it is a biopsychosocial and multicomponent intervention, which includes an initial patient-FP interview, specific FP training and an app.

The app includes a validated risk algorithm to predict depression and a monitoring system that support the DSS for patients and FPs. In the beginning, patients are asked to self-complete a set of questions and questionnaires online. All the information generated from this set of questionnaires is automatically returned to each patient through a comprehensive but brief personalized report, using non-technical language. The FPs also had access to the patient´s report through their website, once the patients had given their consent. The report included individual probability of becoming depressed in the next 12 months and those modifiable risk factors (predictD risk algorithm) for each patient.

Integrating all inputs, the DSS, through pre-designed decision algorithms, proposed a personalized prevention plan (PPP) to each patient from a combination of some of the following eight intervention modules, which had shown some evidence of the effectiveness in the prevention of depression:

- The physical exercise module aims to reduce sedentary lifestyles. The module offers a set of videos for exercising at home but also provides a list of community resources for engaging in physical exercise in each neighborhood.
- The problem-solving module is designed for people who have any type of problem and want to solve it. This module teaches the use of problem-solving strategies.
- The social relationships module aims to improve interpersonal relationships by promoting the uptake of positive group activities. A list of community group activities carried out in the neighborhood is offered.
- The communication skills module teaches patients to develop communication skills by practicing good communication principles with others in a real-life context.
- The decision-making module instructs patients to apply several steps based on decision-making strategies. Patients commit to practicing these strategies in a real-life context.
- The assertiveness module comprises information and exercises to teach people to express their emotions in an appropriate manner, without hostility or aggressiveness. Exercises for coping with difficult communication situations are included.
- The sleep improvement module is directed at persons with sleep problems. This module provides information about good sleep habits and encourages people to follow these recommendations. It also suggests keeping a sleep quality diary.
- The working thoughts module aims to reduce automatic thoughts and worries. This training teaches how to identify and analyze automatic negative thoughts and turn them into positive thoughts. Concerning worries, the goal is to analyze and reduce them through relaxation exercises, mindfulness, psychoeducation and monitoring.

These modules are self-guided, based on principles of cognitive behavioral therapy, have the same structure and are interrelated with each other. They have 1) an initial part on psychoeducation, definitions and motivational messages; 2) an initial evaluation; 3) a pool of recommendations; 4) exercises to apply-practice what was learned from psychoeducation; 5) reminders for working on what was learned; 6) a set of tools (videos, bibliography, links to other modules); 7) a review of compliance with commitments.

Patients were asked to select at least one intervention module; however, there was no limit to using more than one. Once patients chose their own PPP to be self-implemented over the next 3 months, a monitoring and support plan for each specific PPP was established, sending messages, short reports, tracking charts and alerts. FPs could check their patient’s report and the modules on which their patients were working (PPP) at any time.

The PPP was discussed with the FP in a semi-structured 15-minute interview at baseline. FPs were taught how to properly perform the interview. They received a 10-hour training workshop for preparing a personalized depression prevention plan together with the patients and considering patients’ internal resources and clinical data included in their primary care electronic health records. The semi-structured patient-FP interview had the following script: 1) Greet and frame; 2) Open-ended initial question; 3) Active listening about the patient’s risk and protective factors for depression; 4) Detect emotions and empathize; 5) Invite the patient to verbalize attitudes and behaviors that they are already using to prevent depression; 6) Reinforce those attitudes and behaviors that have been scientifically shown to help prevent depression and discourage those that are risk factors for depression; 7) Invite the patient to suggest new attitudes and behaviors to prevent depression; 8) Ask about the personalized prevention plan (PPP) that the app has suggested and help them understand it; 9) Strengthen adherence to the intervention; and 10) Close the interview and communicate your “open door” attitude. For more details of the FP training and patient-FP interview see Bellón et al., 2023 [37].

### Outcomes and assessments

Assessments were completed at baseline and at the 3-month follow up.

The online screening questionnaires were:

1. The nine-item Patient Health Questionnaire (PHQ‐9) [39] which was used as a screening instrument. This questionnaire consists of nine items that evaluate the presence of depressive symptoms present in the last 2 weeks, corresponding to the DSM criteria. The PHQ-9 shows good psychometric properties in Spain [40].
2. The risk-predictD questionnaire is applied to screen the probability of the onset of major depression at 12 months (predictD ≥ 15%). This probability is obtained from the “predictD-Spain” equation that has been previously validated [32]. The full set of items and questionnaires included in the predictD instrument is available in the protocol of the main study [37].

After a positive screening, the patients self-completed the following questionnaires and items through the app:

- The seven-item Generalized Anxiety Disorder (GAD-7) questionnaire which comprises seven items measuring symptoms and severity of anxiety based on the DSM-IV diagnostic criteria for GAD. The Spanish version of the GAD-7 has shown excellent metric properties [41].
- Two questions from the Spanish version of the Duke-UNC-11 instrument to evaluate perceived functional social support referred to affective social support and confidential social support [42].
- Assertiveness using a question about the difficulty of saying ‘no’ to other people (four Likert-type response options).
- Communication using a question about the difficulty of communicating with other people (four Likert-type response options).
- Whether the participants have a conflict or problem with someone and they want to resolve it (yes/no).
- Whether the participants have difficulty making decisions (yes/no).
- Physical activity using the validated Brief Physical Activity Assessment questionnaire for FPs, which consists of two questions, one assessing the frequency and duration of vigorous-intensity physical activity and another assessing the frequency and duration of moderate-intensity physical activity (including walking) undertaken in a week. A score >4 = “sufficiently” and 0–3 = “insufficiently” active [43,44].

At 3 months follow-up the patients self-completed all questions and questionnaires through the app, both the screening and the rest of the assessments.

### Qualitative analysis

A thematic analysis was used [45]. Two analysts were involved (PMP and HCP) to triangulate the information. They listened, read and reread the interviews, and then themes were identified and codified. Later, once they convened to compare and discuss differences in the analysis, themes were recoded and classified, identifying common patterns and convergences and divergences in the data through a process of constant comparison.

The methodological rigor was guaranteed with triangulation, an interview guide, observation, a personal research diary alongside the data collection and contrast of information obtained through the respondent validation technique.

### Quantitative analysis

All the analyses were performed with Stata, version 17 (Stata-Corp). To analyze the differences in scores between pre- and post-intervention symptoms of depression [42] and anxiety [43], probability of the onset of major depression in the next 12 months [32] and mental and physical quality life [44] at 3 months follow-up, we used multilevel linear regression models with two levels: repeated measures nested within participants, and participants nested within family physicians (FPs). As random component was included the FP to account for clustering effects. As a fixed effect, each model included time (baseline vs. 3-month follow-up), and the respective measurements of the dependent variables (symptoms of depression and anxiety, probability of major depression and mental and physical quality life). In adjusted models, we controlled for sex, age, and educational level. Adjusted mean differences and 95% confidence intervals were estimated using the margins command in Stata.

## Results

### Setting and participants

**Fig 1:**
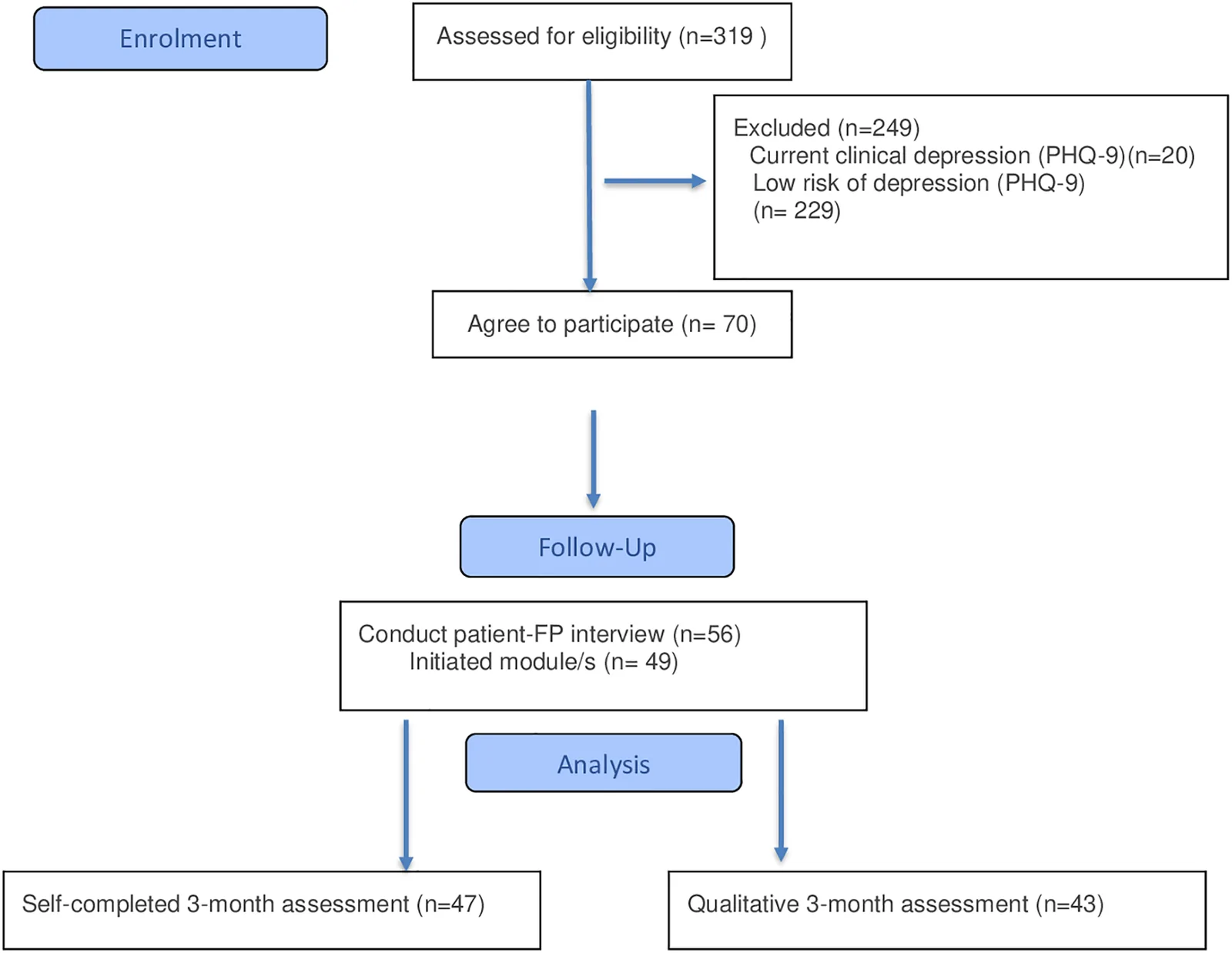
Flow Diagram of recruitment and assessments of primary care attendees included in the e-predictD pilot study.

The study was carried out in six Spanish cities in four autonomous communities. The participating cities were: Málaga, Jaén, Linares, Salamanca, Zaragoza and Barcelona. Six FPs, one from each province, were involved in this pilot study. From these six FPs, 319 patients agreed to participate in the study (see Fig 1). Of these participants, 70 did not have exclusion criteria and met all the inclusion criteria, and 56 carried out the intervention interview at baseline with their respective FP and downloaded the β- version of the e-predictD app; while 47 patients self-completed the assessment through the app at 3 months follow-up (Table 1). The app was utilized for a median of 6 days (interquantile range: 1–30).

**Table 1:** Recruitment and assessments of primary care attendees included in the e-predictD pilot study.

	Málaga	Jaén	Linares	Zaragoza	Salamanca	Barcelona	TOTAL
No exclusion criteria	34	97	100	35	21	32	319
^a^ Excluded	17	85	89	26	10	22	249
Current clinical depression (PHQ-9)	6	1	7	1	1	4	20
^b^ Low risk of depression	11	84	82	25	9	18	229
Agree to participate	17	12	11	9	11	10	70
Conduct patient-FP interview	12	11	9	7	11	6	56(100%)
^d^Initiated module/s	12	7	9	6	11	4	49(87.5%)
Self-completed 3-month assessment	12	6	9	5	11	4	47 (83.9%)
^e^ Qualitative 3-month assessment	9	6	9	5	11	3	43(76.8%)

^a^Excluded for not meeting all inclusion criteria; ^b^ < 15% risk of major depression in the next 12 months (predictD risk algorithm); FP: Family Physician; ^d^ started any module, reviewed at least initial psychoeducation; ^e^ semi-structured interview.

Regarding qualitative analysis, the interviews were conducted with the six FPs and 43 of the 47 patients who self-completed the questionnaires at 3 months.

## Baseline characteristics of the participants

### Patients

The patients participating in this pilot study were mostly women (73%). The mean age was 40 years, with a minimum of 18 years and a maximum of 54 years. Almost 70% of the patients had a college or university education. Half of the patients had a score between 5 and 10 points on the PHQ-9 depression scale and only 16 (28.6%) of the 56 patients had a score of 10 or higher. Regarding scores on the GAD-7 anxiety scale, approximately 60% of the participating patients had a score greater than 10. The probability of “high” or “very high” risk according to the predictD risk algorithm occurred in almost 80% of the patients. Table 2 shows the principal characteristics of the participants at baseline.

**Table 2:** Descriptive patient data at baseline (N = 56).

Characteristics		Patients
**Age (years), mean (SD)**		39.6 (9.7)
**Age (years), n (%)**		
	18-25	6 (10.7)
	26-40	20 (35.7)
	41-5	30 (53.6)
**Gender, n (%)**		
	Male	15 (26.8)
	Female	41 (73.2)
**Education level, n (%)**		
	University	15 (26.8)
	Higher education	24 (42.9)
	Secondary	14 (25)
	Didn’t finish studies	3 (5.4)
**PHQ-9 score, n (%)**		
	0-5	12 (21.4)
	5-10	28 (50)
	>10	16 (28.6)
^a^ **GAD-7 score, n (%)**		
	0-5	2(3.6)
	5-10	20(35.7)
	>10	33(58.9)
**Risk of depression next 12 months, n (%)**		
moderate	(15% −19.99%)	12(21.4)
High	(20% −30%)	18(32.1)
very high	(>30%)	26(46.4)
**Mental quality of life (SF-12), mean (SD)**		39.5(11.4)
**Physical quality of life (SF-12), mean (SD)**		44.4(12.4)

a* 1 missing

### Family physicians and Health centers

The FPs who participated in the pilot trial included three women and three men with a mean age of 59 years (SD: 4.5). The study was conducted in six PCCs in four Spanish Autonomous Communities (Andalusia, Castilla y Leon, Aragon and Catalonia). The average population served by the health centers was 28,829 people (SD: 15992). The number of physicians in each health center ranged from 8 to 20, with an average of 13.3. The number of pediatricians in each health center ranged from 2 to 4, with an average of 2.8. The number of nurses in each health center ranged from 8 to 19, with an average of 13.3.

### Qualitative results

See Fig 2

**Fig 2:**
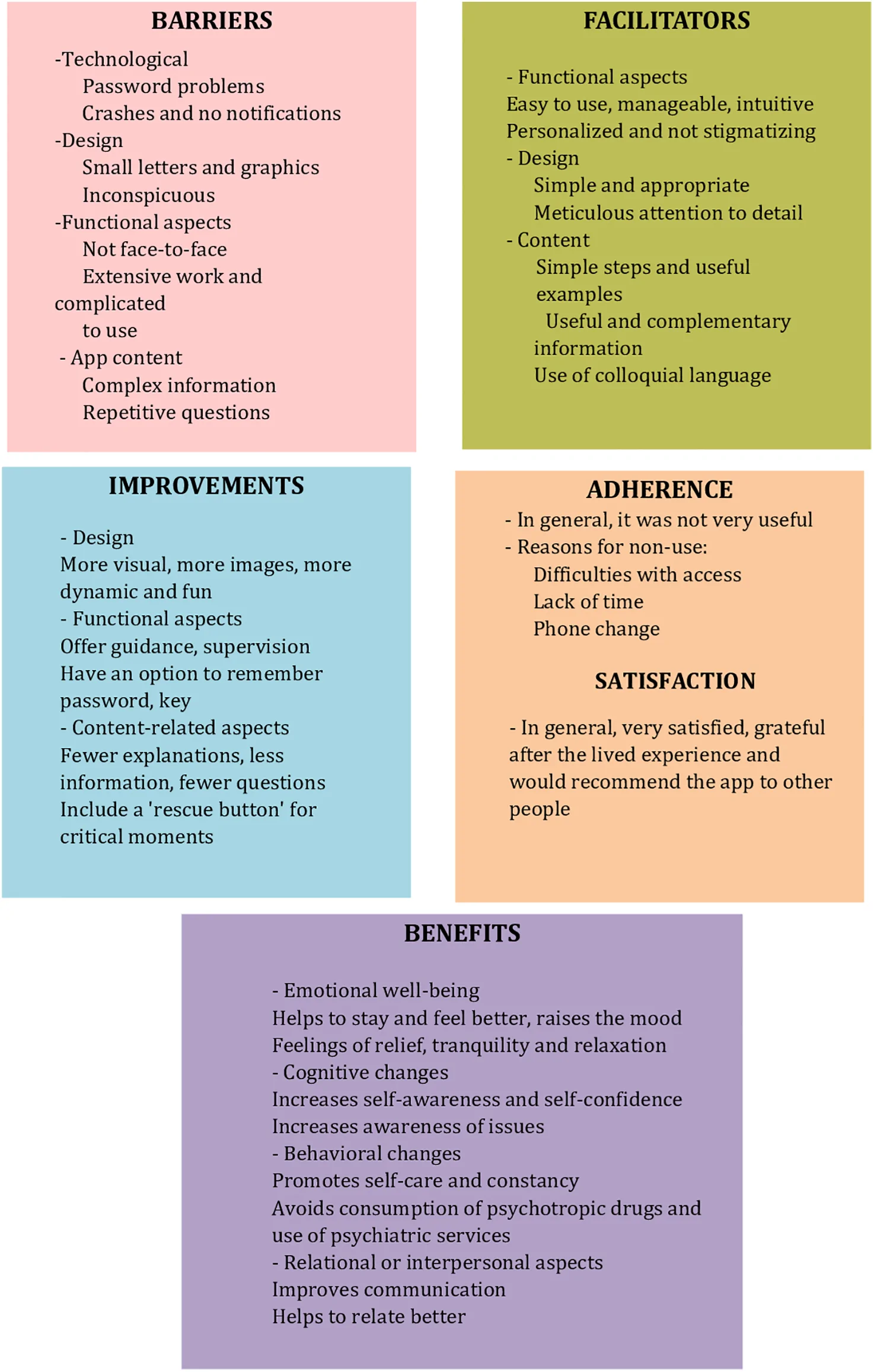
Summary of qualitative results.

#### Patients’ opinions.

Patients in the e-predictD pilot study identified different opinions regarding their participation in the study and their experience using the e-predictD app. These opinions were classified into the following categories: usefulness-benefits of the app, usability of the app, modifications of the app and evaluation of the interview with their FPs. A summary of the qualitative results is shown in Fig 2.

1. Perceived usefulness-benefits of the e-predictD app

In general, e-predictD app users identified different beneficial aspects of the app in particular and the e-predictD intervention in general.

1.1. Emotional well-being

According to the opinion of the participating patients, it helps them to stay and feel better, it raises their spirits. It also provides relief, tranquility and relaxation, helping to manage discomfort and reduce fears.

(2*: I replied, in this case, that I felt more relaxed and calmer*). (22: A*nd then I started to feel more like doing more things. Your mood is more alert, more cheerful. More of saying I want to do this and that and you no longer see limits. You yourself, let’s say that you conquer some fears, it’s about overcoming those fears. It really helps*.)

1.2. Cognitive changes

Regarding cognitive aspects, patients reported a number of changes such as increased self-knowledge and greater awareness of problems.

(32: *To me the truth is that I found it very positive, because it helped me to get to know myself better and to ask myself new questions*… *to reflect more on some… specific things*) (28: *But it did make me think about things and it did make me see that I was, that I was at risk and to realize*)

Some patients also commented that using the app helped them to be more positive and contributed to an increase in self-esteem and confidence.

(8: *all these guidelines*…*the reinforcement always towards the positive, you know? I was a person who always saw the bottle as half empty, so I always*…*in the end I said that they always lead you to the bottle half full.*) (13: *Well, in self-esteem I think more positively, I’m very negative*…*And so I think more in*… *in a positive way.*)

1.3. Behavioral changes

According to the participating patients, the application helped them to take care of themselves, sleep better and do more exercise, among other things.

(5*: Maybe changing habits that I had… more doing sports and the truth is that now I feel much better.*) (41*: Well, in learning to solve this type of… problems or circumstances that come up and in improving my routine habits, both in physical exercise and in setting certain goals…*) (42: *yes, it has helped me with my sleep and I am a little more regular when it comes to exercise…*)

Some patients felt that it helped them to work on assertiveness and to communicate better with other people.

(41: *For me, especially the part… of assertiveness, how to express myself, how to handle a problem and how to make another person see it in a very simple way without… without shouting, without all that, for me it was all those indications, for me they are already my values, I mean… it has really helped me a lot… Assertiveness I didn’t even know what it was and for me it has been key in… my recovery and my approach in my new stage and mmm how to communicate with other people, because I needed it*).

It also benefited them in solving problems and improving personal relationships.

(5: *Ehhh…. When it comes to talking to people or relating more… I have always been very reserved and never spoke and maybe I felt more closed and with the application, seeing what it said, I have opened up more. And also in terms of conflicts with other people, knowing how to handle yourself)* (12: *And if it also helps you to resolve a problem, of course it’s a useful application. There are twenty thousand applications that are not useful, but this one is.*)

Finally, some patients reported that the app was useful for their entertainment.

(7: *Well, if some people… feel lonely, or have to… well, it entertains you a lot)* (24: *It keeps you entertained and makes you read more, yes)* (34: *Well, let’s see, the biggest problem is anxiety, but since I am very nervous by nature, well, it helped me, because it entertained me and I had to do things, so I was entertained*).

2. Factors than facilitate the use of the e-predictD app
2.1. Functionality

Patients indicated that the app was intuitive, manageable, understandable and easy to use. They appreciated that it offered flexibility and freedom in its use.

(14: *It’s easy it’s very easy… anyone can use it, even older people…the appearance, the way of expressing the types of exercises, they are not complex at all) (15: it’s an application that guides you by itself…it’s easy and simple I mean it’s not an application that is hard to use). (8: that you can use it freely from your home, whenever you want and as freely as you want, for me it’s great)*

The reminders were also greatly appreciated as they helped some of the participants to plan how and when to do the work.

(16: *it offered you the possibility… to remind you and to ask you how you were going to do it… it reminded you that you were working on those points and that you were in a certain section and that you had to come back in a week… And in a week you came back and “hey, let’s go on to the next step. Did you do what you had to do? Yes, no… well, let’s move on” and so on. Yes, I liked that aspect a lot*).

2.2. Reduces stigma

According to the participants, as it is an app on the phone, it reduces the still existing stigma surrounding mental health and using mental health services.

(5: *Sometimes we need to be given some guidelines or counseling… and maybe you don’t want to go to a person for counseling because of embarrassment or whatever, and you have it on your mobile phone. It is easier*.) (29: *It explains things well and it is more freeing because you enter, as nobody asks you, nobody tells you, so you enter what you want, what is really happening to you*).

2.3. Content

Users valued as positive the fact that the app offered a personalized plan and that it was tailored to the person’s needs, even noting that it was similar to having a specialist at their side, who cared about their state of health.

(1: *Well, because it also offers you a personalized plan based on the answers you provided to the questions… So that helps you a lot to see what… the problems you have and how you can solve them.*) (11: *it’s like having the psychologist there in your house telling you: “now do this, and try to do it like this, I can help you like this, let’s do it like this” for example, for me in my work situation it has helped me a lot because it shows me the pros and cons*) (42: *you feel like there is someone who listens to you and also if you have the possibility of… of having these tools or a doctor who also understands you then… it is very good*).

The tips, resources and links provided by the app were valued as useful in situations where they were needed.

(31: *I found them to be interesting tips that I could apply to my daily life or to the problems I had*). (15: *I liked the application itself, because, in addition, apart from what the application itself is, it directs you to several sites to various links to the understanding of mindfulness, the studies conducted by the University*).

Finally, the fact that the FP was also connected to the application was positively valued.

(42: *the fact that you have someone so close to you, who knows your problems and who is also familiar with the application, who can give you recommendations… from within the application or medical advice is quite… quite complete*).

3. Perceived barriers to use of the e-predictD app
3.1. Technology

Regarding the technological aspects of the app, patients identified problems with the password, they had difficulties remembering and entering it and, in addition, the system failed to recognize it. They also pointed out that the app crashed, could not be used, and, in some cases, notifications did not work correctly since sometimes they were not received and sometimes they appeared all at once.

(38: *although I saved the login information, many times it doesn’t recognize it, so I have to wait a while or at that time it does not let me enter*). (10: *you get errors multiple times and there were some days that I couldn’t use the application*) (5: *the notifications were not working, so I didn’t get them and one day I would get fifty notifications in a row*).

3.2. Content

Regarding the content, patients pointed out the complexity of some of the information and questions provided by the app. In addition, they considered that both the information requested and returned by the app was excessive and time-consuming.

(15: *There were some questions that I didn’t really understand what they were asking me*) (6: *P: I think there are too many questions, but I do get that it is to try to find out exactly what is going on with you*) (35: *I think it’s good, but there are times when I thought there was too much information. I mean, too much information, I think it should be shortened.*

Some people did not have confidence or credibility in the app’s prediction of depression risk. They commented that it was shocking and even worrying to be told that they might have depression in the future, when they also consider that the circumstances that determine such predictions can change rapidly.

(26: i*t depends a lot on the circumstances, it depends on the moment, it depends, because of course when a family member dies, it is logical to be sad, but that does not mean that you are going to be depressed. But it is true that during the last four or five weeks, well, maybe you’re sad, but that does not mean that you will have depression. So, it is very relative, it is very relative*) (29: *Well…the information. It told me that I had a high risk of depression and the truth is, I think that right now I don’t have that high risk, but maybe it was because of the answers I gave…I don’t know how it will be evaluated and so forth. That was what shocked me the most*).

In some cases, users reported feeling overwhelmed by all the reminders and alerts sent by the app, and some people commented as a negative part of the application that they had felt obliged to commit to dates and times when this was not helpful to them.

(19: *I mean, it was more useful for me to write and to have personal life guidelines than to be forced to “come on, think about what day you have to meet and think…”. No, no… it didn’t help me. That is a negative part of the application*) (31: *that maybe for some modules it was very insistent because you had to have a schedule of every day*)

3.3. Design

Regarding design, participants noted that the font and graphics were too small.

(26: *very small letters*) (26: *There were graphics that gave you examples and I tried to enlarge the screen like on mobile phones…And you couldn’t see it*)

4. Modifications and improvements to the e-predictD app

The patients participating in this study suggested a series of potential improvements to this app related to the design and functionalities of the system such as having access to everything that the person includes in the app, having some type of guidance or supervision and including audios or videos so that the person does not have to read so much.

(7: *Well, more visual and less letters, yes) (5: that you can always see what you wrote before*) (15: *but maybe a little bit supervised too, because at a given moment there might be someone who has a doubt, has a problem…*) (43: *I don’t know, maybe some video could be added or something that, I don’t know, because they would do a little bit more)*.

In addition, they proposed including challenges and prizes to make it more fun to use.

(16: *Or like a test or… something simple because we don’t want them to say “this is boring… at least I’m very… I really like these addictive games and stuff*).

Regarding the contents of the app, the participants suggested fewer explanations, less information, and if the person wants to know more, that it should offer links to further information.

(9: *… a little less reading so that the questions and so on don’t become so long. It’s good because when you answer a question they clarify it, you know?… I think it would be better to make it a little bit shorter*) (40: *something more… like… the information that provided a heading of what it is, and then maybe if I am interested in going into it further, with a more extensive text, with some exercise, maybe, I don’t know, or… some reference that is easy to understand or a link to a website that… that explains a little bit more at length how to approach this type of problem or I don’t know, something like that*).

In addition, they proposed reducing questions and presenting them in a simpler way.

(39: *Maybe it’s just that some modules did not ask as many questions or as often.*)

5. FP-patient interview

The patients valued this interview as a moment when they felt at ease, comfortable and felt very close to their physician, partly because there was a previous relationship of trust.

(12: *Everything was very correct, very personal and I was very comfortable*) (22: *… I felt very at ease*) (30: *Well, since I trust her so much, she’s been my family professional for many years now, it feels as if we know each other very well, very close… It’s as if we look at each other and understand each other and I don’t know, I felt very comfortable with her and everything, I felt very good, very good, to tell her my stuff*)

The patients appreciated and considered it important that the FPs showed interest and took the time to address this issue.

(6: *Well, the truth. Ummm they ask you questions, I see that they take the time with you to find out… that is very important that they take the time*) (10: *well, the interview with the doctor was good, I saw that the doctor was interested, and was concerned about my particular case*)

The patients also felt that this interview helped them talk about more aspects of their lives.

(2: *Exactly. I talked more with her*) (24: *maybe in a normal consultation, when I see her, we don’t talk as much, so that interview was good for me*) (40: *it also gave me the opportunity to talk a little bit more about… what my problem is*).

#### FPs’ opinions.

The FPs participating in the e-predictD study identified different opinions regarding their participation in the study and their experience using the e-predictD platform. These opinions were classified into the following categories:

1. Facilitators of the use of an m-Health tool
1.1. Openness to the use of new technologies, in the sense that its use is also easy and the structure and content is good:

(5: *Well, I thought it was an interesting experience, given that studies are now focused on the use of new technologies to diagnose and treat patients*) (2: *what is really good about the platform is that it structures the work and explains it to you in a clear and understandable way* (2**:** *Offering a tool within the reach of the user, of the patient who has it on their mobile phone, and that will help them in their day-to-day activities, in their life….*) (6: *… it seems to me that it is going to be a very important tool for the family physician, and for a society that is increasingly medicalized, increasingly frustrated with a low threshold, all pills, all immediate fixes, I think that giving them tools seems to me to be a high priority, in my opinion*)**.**

1.2. Openness to the approach to depression prevention and improved communication with FPs. The app facilitates the approach to depression and communication with the FPs.

(1: *I found it interesting and appealing and, well, being able to incorporate this tool for some people who did not take the step to seek or not seek help to prevent depression, well, we encountered a lot of people who found it interesting*) (4: *Well, my experience as a doctor, positive, because the truth is that at first the patients experienced it as an opportunity to improve communication with me*) (2: *To be honest, using the platform with patients opens up some dimensions of preventive work that we do not have integrated in the clinical history and that with the platform you can use more easily, so in this sense it is a help, yes*) (5: *Yes, you already know what to do when there is depression or anxiety, but what to do or how do you know if this patient is at risk of depression? Well, the truth is that we do not explore that. When the task of the family physician should be above all preventive tasks and activities, then also when you are familiar with the tool, well, you can use it, of course, knowing that it exists, which until now I did not know about it*)*.*

1.3. It contributes to the empowerment of patients and to the change of the patient’s role from a passive to an active subject.

(1: *Well, for some of them it has made it easier in the sense that they have pushed themselves to take more self-care, to dedicate more time to themselves and to take care of themselves,…, they have found in the app a reason to push themselves and to make plans, it gave, so to speak, a wake-up call, an alert*)*.* (4: *Let’s see, what I liked about the app is that it offers proposals for activities…, well basically, activities and things that the patient can do… that help them to improve their self-knowledge and also to become more empowered in taking care of their health*) (5: *what the app does is to involve the patient in their own therapy, so of course they have to do something, you support them, but they are the ones who have to do it*)*.* (6: *the preventive vision, the approach that encourages the patient or their environment to apply resources that they have or can have for themselves or their close environment, to avoid medicalization or pharmacology in more adaptive situations.*)

1.4. Provides valuable information for the FP

(3: *As a physician… uh… I do. I think that yes, I was able retrieve what I was interested in at all times. What interested me, of course, was the report on each of the questionnaires and tests, in order to have a picture of the risk profile, which are the aspects that will help me to make decisions*) (4: *As a professional, as I said, it gave me very valid information about my patients that I might not have otherwise, about the life events they have had, about their risk of suffering from depression… I found it interesting… their anxiety levels, their levels of depressive symptoms… I think it is relevant information for a doctor*).

1.5. In addition to the app, the fact that this intervention was also based on a face-to-face FP-patient interview facilitated the opportunity to get to know the patients.

(4: *And above all for me it was an opportunity to get to know my patients better, the interview we did with them I really discovered that despite the fact that I had been treating them for some time, there were aspects of their health and their emotional health that I was not aware of and of their personal circumstances… It gave me more opportunity, perhaps, to get to know the patients better.*).

1.6. Ease of use

(5: *The appearance was good, it was colorful, the font size was good, I think it was simple, yes, it was quite practical and very accessible to everyone… Yes, yes it was very intuitive, yes. It was quite practical and very accessible to everyone. Even if they had little knowledge of computers it was very simple, in fact, most of them knew how to use it well.*).

2. Barriers to using an m-Health tool
2.1. Technical difficulties

(4: *I did not like it, perhaps the technical difficulties we sometimes had with the patients either to download the application or to enter… well, I don’t know… technical difficulties. I suppose that everything can be improved* (4: *To begin with, logically, the technical aspects: that it is not difficult to download it, that it does not require the patient to come two or three times until they manage to download the application, that it does not freeze, that their mobile phone does not have to be a special type of phone* (1: *the main complaint in some cases was the accessibility when filling in the PIN, as the screen was cut off and it was difficult, and then to look for the little symbol, the bracket, some complained that it was very difficult to start it… we commented on some incidents, starting with the initial accessibility of the PIN.*).

2.2. Inability to manage the tool and patients

(5: *The truth is that the modules were already predefined and I did not really find a way to remove the ones I did not need. I could add, but if there was one that I wanted to remove, the truth is that I could not remove it… yes, they were related to the problem they had, but I would not have put so many of them, in fact, I told them to focus on this one and this one, in particular*) (6: *only I knew when they had done the questionnaires, which modules they had chosen and that they were going to start working on them, but I didn’t know if they got involved, if they didn’t get involved, if they used community resources from the map that I prepared, so I’m not clear at this point how much it helped them… that I could simply look as if in the background if they were making use of what we had agreed a priori that they were going to try to do*) (1: *I didn’t know the contents of the platform itself and how it updated or how it interacted with each of the users, so, well, I was a little bit between uncertainty and curiosity and I missed the fact that the professionals had access to the specific contents of the platform. I think that, as professionals who recommend this, the strategies that we have, and especially if we want to disseminate the app among users, among professionals, then at least they should have access to it so that professionals can be trained and know exactly what recommendations we are making for this type of therapy.*).

2.3. Requires interest and time on the part of the patient.

(4: *The difficulty is that it is not something that is given and that patients receive in a passive way, but that they have to take time and interest to use it and to get into it and to manage it*) (6: *Another thing was the difficulty to find time to read it slowly, to fill it in and then to put it into action*).

2.4. Difficult to understand information

(5: *sometimes there was contradictory information, so sometimes it was not very clear to me, maybe it was the way it was expressed*) (3: *However, I have the impression that patients still do not understand the reports very well… They have a general idea, but when you ask them…they only know if they are doing well or not and little more…*).

3. Future improvements
3.1. Involve more professionals.

(6: *I also believe that social workers should be more involved, I…how to better optimize the social worker in your area, who, for me, is an important link, in this instance, with our patients.*).

3.2. Mode of communication

(2*: but maybe we should have a more positive approach in terms of wellbeing; talk more about wellbeing and less about disease, but this is my view*) (3: *maybe we should improve the way information is given and how to retrieve information about the patient, make it more user-friendly*).

3.3. Quantity of information: fewer questionnaires and assessments

(4: *Perhaps not burdening the patients with questionnaires that they are not sure are really of benefit to them. I would try to ensure that the tasks for the user are aimed at improving their health problems rather than at evaluating services and resources.*).

### Quantitative results

The β-version of the e-predict-D app was downloaded by 56 participants. The mean number of recommended modules was five (SD 1.90). The most recommended module was the working thoughts module, 53 (94.4%).

Of the patients who were recommended the working thoughts module, 72% worked on it, with this being the most worked on module. (Table 3). The results indicated low levels of user engagement.

**Table 3:** Usability of the β-version of the e-predictD app (N = 56).

Intervention Modules	Recommended, n (%)	^a^Worked on, n (%)
Communication Skills	22 (39.3)	9 (39.1)
Assertiveness	29 (51.8)	12 (41.4)
Decision-Making	42 (75)	12 (29.3)
Physical Exercise	39 (69.6)	20 (52.6)
Working With Thoughts	53 (94.4)	39 (72.2)
Social Relationships	29 (51.8)	9 (31)
Problem-Solving	44 (78.6)	11 (25)
Improving Sleep	22 (39.3)	14 (56)

^a^Intervention modules worked on of the total recommended

At 3 months follow-up, the patients participating in the e-predictD pilot study showed a statistically significant decrease in risk of depression in the next 12 months, anxiety symptoms and increase in mental quality of life. However, there was no significant reduction in depressive symptomatology or improvement in physical quality of life (Table 4). No adverse effects were reported in the open-ended sections of the semi-structured interviews.

**Table 4:** Health outcomes in the e-predictD pilot study (N = 56)*.

Health outcomes	Mean at baseline (95%C.I.)	Mean at 3 months (95%C.I.)	Mean difference (95%C.I.)	P
Depressive symptoms (PHQ-9)	8.76 (7.02 to 10.50)	9.63 (7.77 to 11.49)	0.87 (−1.68 to 3.42)	0.503
^**1**^ Depressive symptoms (PHQ-9)	8.81 (7.03 to 10.60)	9.62 (7.73 to 11.51)	0.80 (−1.81 to 3.42)	0.547
^**2**^ Depressive symptoms (PHQ-9)	8.88 (7.20 to 10.56)	9.58 (7.81 to 11.35)	0.70 (−1.76 to 3.15)	0.578
Anxiety symptoms (GAD-7)	11.38 (10.10 to 12.66)	9.25 (7.87 to 10.64)	−2.13 (−4.01 to −0.24)	0.027
^**1**^ Anxiety symptoms (GAD-7)	11.49 (10.24 to 11.74)	9.13 (7.77 to 10.48)	−2.37 (−4.23 to −0.50)	0.013
^**3**^ Anxiety symptoms (GAD-7)	11.68 (10.33 to 13.03)	9.09 (7.65 to 10.53)	−2.59 (−4.59 to −0.60)	0.011
Risk of major depression at 12 months**	0.345 (0.289 to 0.400)	0.262 (0.202 to 0.322)	−0.082 (−0.165 to −0.001)	0.048
^**1**^ Risk of major depression at 12 months**	0.349 (0.293 to 0.404)	0.258 (0.197 to 0.318)	−0.091 (−0.174 to −0.001)	0.031
Mental quality of life (SF-12)	36.5 (33.7 to 39.4)	43.0 (39.9 to 46.1)	6.50 (2.29 to 10.70)	0.002
^**1**^ Mental quality of life (SF-12)	36.3 (33.5 to 39.0)	43.4 (40.3 to 46.4)	7.08 (2.97 to 11.19)	0.001
Physical quality of life (SF-12)	44.4 (41.3 to 47.4)	42.3 (38.9 to 45.7)	−2.05 (−6.62 to 2.51)	0.378
^**1**^ Physical quality of life (SF-12)	44.2 (41.2 to 47.2)	42.5 (39.2 to 45.7)	−1.70 (−6.11 to 2.71)	0.450

* Multilevel linear regression adjusted for repeated measurements over time and Family Physician as random components.

** Probability of the onset of a major depressive episode at 12 months assessed by the predictD risk algorithm

^**1**^ Models were adjusted for sex, age, and educational level.

^**2**^ Models were adjusted for sex, age, educational level and anxiety symptoms (GAD-7) at baseline.

^**3**^ Models were adjusted for sex, age, educational level and depressive symptoms (PHQ-9) at baseline.

## Discussion

### Principal results

In general, the patients and FPs had a positive perception of the β-version of the e-predictD app regarding its content and personalization. They also positively evaluated the patient-FP intervention interview, noting good communication characterized by sufficient time, active listening and empathy. Patients found the e-predictD app useful, likely due to perceived improvements in their well-being and observed positive cognitive, behavioral, emotional and relational changes. However, they also identified the need for modifications and enhancements in its design and functionality for the final version of the e-predictD app to be developed. Customizing the protocol, by itself, was not enough to reach the targeted engagement levels, as no clear solution to this challenge has yet been established. Overall, patient engagement and app utilization were low, attributed to difficulties in access, device changes and time constraints. Some patients reported feeling overwhelmed by the abundance of content, questions, notifications and tracking features.

### Strengths

Although our research group had already developed and evaluated a personalized intervention to prevent depression in primary care [33–35], this is the first time it has been implemented and tested in a pilot study incorporating an innovative app that includes risk prediction algorithms, personalized depression prevention plans and decision support systems for patients and FPs.

### Limitations

Several limitations are evident in this study. First, due to the absence of a control group with random assignment, we cannot definitively attribute the improvement in outcomes at 3 months to the intervention. Second, the findings are based on a small sample of primary care attendees: predominantly women aged 18–55, with a high risk of depression and anxiety comorbidity, and varying levels of technological literacy. This restricts the generalizability and external validity of the results. Moreover, the FPs participating in this pilot study likely possess higher motivation and psychosocial orientation, further limiting external validity. Third, the FPs were not blinded to the assignment of patients to the intervention and were involved in the recruitment process. Although recruitment followed a strict protocol (with participation offered to all eligible patients daily), there is a possibility of conditioning or bias in the selection process, potentially influencing the participation of certain patients and introducing selection bias. Fourth, the β version of ePredictD was used less than expected, which hampers its effectiveness. As indicated by the qualitative results, difficulties such as access to the app due to password issues or long texts highlight the need to modify the final version to increase its utilization. Fifth, “The requirement of 10 hours of training for family physicians could represent a barrier to implementing this intervention in real-world primary care settings. However, in the qualitative analysis of perceived barriers among participating family physicians, no concerns related to this aspect were identified. It is also possible that the family physicians who participated in this pilot study, compared to those who did not, were more motivated to engage in this type of interview and had a professional practice more oriented toward the psychosocial aspects of care. Furthermore, based on a cost-effectiveness analysis from our most recent RCT, in which the same training for family physicians and very similar intervention interviews were used, we found that even after including the training costs, the intervention was clearly cost-effective for the prevention of depression in primary care”.

### Comparison with prior work

To enroll in our pilot study, patients were requested to select at least one of the intervention modules recommended in their personalized prevention plans. They had the option to utilize as many modules as they wished from the recommended list. We anticipated a higher overall usage of the app, with a median usage duration of approximately 45 days; however, the median was of 6 days (interquartile range 1–30); therefore, the app’s usage was low for us. On the one hand, some patients might have initially been encouraged to use the application by their FPs, but later, since this was an unguided intervention, the likelihood of low use and dropout could have increased. On the other hand, as this was the first version of the app, the password recovery and monitoring functionality had some flaws, which might have made it challenging for certain users to access the app later on. Additionally, excessive text and minimal gamification elements could have been tedious or unengaging for some patients. Finally, from a different perspective, the low usage of the app may be attributed to some patients having already achieved their personal treatment goals before completing the entire intervention.

Internet and mobile device-based mental health interventions have great potential to improve the mental well-being of the population while also reducing the economic burden of mental illness [9,21–23,46]. However, a considerable proportion of users fail to adhere to these interventions [47,48]. Usability is a potential challenge across most of internet-based psychological interventions, as underscored by the results of this pilot study. Achieving robust engagement and adherence is a key challenge for digital mental health researchers. Additionally, unguided interventions typically exhibit lower levels of adherence than guided interventions [49,50], with completion rates often associated with therapeutic guidance [51–53]. In our study, although the FPs recommended the program and collaborated with patients to select the most appropriate and tailored intervention modules, the preventive program was entirely self-administered. Technological issues and time constraints emerged as the most common reasons for low utilization. It is possible that, due to social desirability, patients did not express that the program was boring or tedious. Gamification has garnered significant attention among researchers, with considerable efforts directed towards creating engaging and motivating programs [54–56]. Nevertheless, gamification has not consistently demonstrated a correlation with the adherence to or effectiveness of digital interventions, either in mental health or in general [55,57].

Personalization is a promising approach in the prevention of mental health problems [58]. Our results could support this conclusion. However, different aspects would be included within the concept of personalization of depression prevention. In a depression prevention study, adolescents were classified as high or low on cognitive and interpersonal risk factors and randomized to either a cognitive-behavioral or an interpersonal prevention program. Matched adolescents showed significantly greater decreases in depressive symptoms than mismatched adolescents, although there was no significant difference in rates of depressive disorders [59]. Another study of personalized telephone-based coaching to prevent depression in farmers noted that that there are no set procedures or standardized manuals for the individual coaching process [60]. In a systematic review and component network meta-analysis using individual participant data to personalize internet cognitive behavioral therapy (iCBT) for depression, Furukawa et al. [61] propose combining elements of iCBT based on their results. For example, for a 45-year-old female patient, in a relationship, and with a baseline PHQ-9 score of 12, the best therapeutic result would be achieved by combining the following components in the iCBT intervention: psychoeducation, behavioral intervention, and problem solving, and using automated and human encouragement [61]. Our proposal for personalized depression prevention is built upon the promising results of a previous intervention [33–35] and entails a combination of eight components or modules tailored to each patient. This customization of components relies on decision algorithms integrated into the app. For instance, if a patient exhibited sedentary behavior, suffered from insomnia, and was at high risk of major depression in the next 12 months, they would be advised to engage in physical activity, improve sleep patterns and address cognitive restructuring aspects through targeted modules.

## Conclusions

The β-version of the e-predictD intervention shows promise in terms of feasibility and acceptability among FPs and patients, although patient usability of the app was poorer than anticipated. Patients and FPs proposed several enhancements to the design and development of the final version of the app, which could improve its usability and acceptability. In addition, while improvements in anxiety symptoms, depression risk score and mental quality of life were noted, there was no improvement in depressive symptoms or physical quality of life. This indicates the need for confirmation through a randomized controlled trial.

## Supporting information

S1 Protocol EpredictD protocolo. (PDF)

S1 Checklist TREND Statement Checklist HC26. (PDF)

S1 File Graph. (DOCX)

## Abbreviations

FP: Family physician
ICT: Information and communication technologies
DSS: Decision support systems
PPP: Personalized prevention plan
PHQ-9: The nine-item Patient Health Questionnaire
SF-12: Health–related quality of life using the 12-item Short Form
GAD-7: The seven-item Generalized Anxiety Disorder
