# Supplementary material for: Toward personalized interventions for preventing depression in primary care: Qualitative and quantitative findings from the e-predictD pilot study
Source: PLoS One. 2026 Sep 11;21(9):e0355675. doi: 10.1371/journal.pone.0355675 (PMC13567784; doi:10.1371/journal.pone.0355675)
Supplement: Supplementary file 3 [file pone.0355675.s003.docx]

Health outcomes in the e-predictD pilot study (N=56)

|  | Risk of major depression | Mental quality of life (SF-12) |
| --- | --- | --- |
| Mean at baseline (95%C.I.) | 34 | 36.3 |
| Mean at 3 months (95%C.I.) | 25 | 43.4 |

Models were adjusted for sex, age, educational level

Risk of major depression at 12 months *p* = 0.031

Mental quality of life (SF-12) *p* = 0.001

Health outcomes in the e-predictD pilot study (N=56)

|  | Anxiety symptoms GAD7 | Depressive symptoms (PHQ-9) |
| --- | --- | --- |
| Mean at baseline (95%C.I.) | 11.68 | 8.88 |
| Mean at 3 months (95%C.I.) | 9.09 | 9.58 |

Models were adjusted for sex, age, educational level

Depressive symptoms (PHQ-9) *p* = 0.578

Anxiety symptoms (GAD-7) *p* = 0.011
